# Assessing Awareness and Actions Must Be Taken in Acute Myocardial Infarction: A Cross-Sectional Study on the General Population in Alahssa, Saudi Arabia

**DOI:** 10.7759/cureus.49300

**Published:** 2023-11-23

**Authors:** Abdullah Almaqhawi, Abdullah Alkhalaf, Mohammed Al Qadhib, Ali M Alhashim, Norah S Alsaad, Mshari S Alqahtani, Mohammed Alqahtani, Osama F Alamri, Fatimah Buali, Ibraheem Alhusain

**Affiliations:** 1 Family and Community Medicine, King Faisal University, Hofuf, SAU; 2 Internal Medicine, King Faisal University, Hofuf, SAU

**Keywords:** myocardial infraction complications, awareness myocardial infraction, awareness, myocardial infraction, acute myocardial infraction, ami

## Abstract

Introduction: Acute myocardial infarction (AMI), commonly referred to as a heart attack, is a leading global cause of mortality, necessitating immediate recognition and appropriate actions. This study aims to assess AMI awareness and response among the general population in Alahssa, Saudi Arabia.

Methodology: A descriptive cross-sectional design was employed, with surveys distributed via online platforms. Participants aged 18 years and older, residing in Alahssa, and providing informed consent were included.

Results: Among 406 participants, 74.1% had good knowledge levels. Notably, 216 (53.2%) recognized calling an ambulance as the initial response to AMI symptoms. The Internet (140, 28.9%) and healthcare professionals (113, 23.3%) were primary information sources, with "sudden pain or discomfort in arms or hands" (287, 27.8%) as the most recognized symptom. "Obesity" and "heart disease" (304, 12.3%) were the most common risk factors. Demographic variations in knowledge were observed.

Conclusion: This study highlights the need to enhance public awareness of AMI, particularly among specific demographic groups. Targeted educational campaigns can improve knowledge, promote appropriate actions, and ultimately enhance outcomes during AMI events in Alahssa, Saudi Arabia.

## Introduction

Acute myocardial infarction (AMI), commonly known as a heart attack, is a life-threatening condition characterized by the sudden blockage of blood flow to the heart. It is the leading cause of death globally, accounting for three million deaths each year and one million in the United States [1]. The prevalence of AMI has increased in Saudi Arabia in recent years, reaching 5.5% overall [2]. AMI is associated with various risk factors, including hypertension, smoking, hyperlipidemia, diabetes, and a family history of coronary artery disease [3]. Additionally, factors like obesity, a sedentary lifestyle, and excessive alcohol consumption are linked to an increased risk of AMI, and advancing age is also a significant risk factor, with older individuals being more susceptible to AMI [4]. AMI can result in a range of complications, including arrhythmias, heart failure, cardiogenic shock, and, in severe cases, even death. Ventricular remodeling may occur following AMI, leading to long-term cardiac dysfunction [5], and post-infarction angina and recurrent myocardial infarctions are possible complications that can arise after the initial event [6]. Immediate management of AMI includes aspirin, nitroglycerin, and oxygen to alleviate symptoms and prevent further damage. Primary PCI is the preferred method to restore blood flow in the blocked artery; if not available, thrombolytic therapy like tPA can be used to dissolve clots and restore heart muscle blood flow [7]. Early recognition and prompt initiation of appropriate actions are crucial in minimizing the extent of myocardial damage and improving patient outcomes [8]. Despite the availability of educational campaigns and advancements in medical technology, studies have shown that there is still a lack of awareness and knowledge regarding AMI symptoms and the necessary actions to be taken in case of an emergency [9-10]. Moreover, a study conducted in Saudi Arabia showed that the level of awareness regarding AMI was suboptimal [11]. This knowledge gap poses a significant challenge to the timely initiation of appropriate medical interventions, potentially leading to poorer patient outcomes. To address this issue, we conducted this study to assess the level of awareness of AMI and the first action that should be taken in case of an AMI episode among the general population. This population-based study aims to gather comprehensive data on public awareness in Alahssa, Saudi Arabia.

## Materials and methods

Study design

This study employed a descriptive cross-sectional design to assess the level of awareness of AMI and the first actions taken in case of an AMI among the general population. Surveys and questionnaires were utilized to capture information on participants' awareness of AMI symptoms and their knowledge of appropriate actions to be taken during an AMI episode.

Study settings

The study was carried out in Alahssa, Saudi Arabia, from October 1, 2023, to November 3, 2023, by using an online survey distributed on social media platforms.

Inclusion and exclusion criteria

The study targeted the general population, aiming to gather a representative sample of individuals with varying demographic characteristics, such as age, gender, socioeconomic status, and educational background. The inclusion criteria for participants involved individuals aged 18 years and older, Saudi citizens living in Alahssa, and those who provided informed consent to participate in the study. Certain individuals were excluded from the study, including non-Saudis not residing in Alahssa, individuals with known cognitive impairments or communication challenges that could hinder their ability to provide accurate responses, and those who couldn't understand the questionnaire due to language barriers.

Data collection tools

The questionnaire was designed specifically for this study, comprising a series of structured questions addressing the study's objectives. The tool included sections to assess participants' knowledge of common AMI symptoms, their understanding of the urgency of seeking medical help, and their awareness of the appropriate actions to be taken during an AMI episode.

Data analysis

The data was collected from the questionnaires and analyzed using SPSS Statistics version 22 (IBM Corp. Released 2013. IBM SPSS Statistics for Windows, Version 22.0. Armonk, NY: IBM Corp.).

Ethical considerations

The study complied with all relevant privacy and data protection regulations, including the General Data Protection Regulation (GDPR) guidelines. Any identifiable participant information was stored securely and accessible only to authorized personnel. The study also adhered to ethical guidelines established by the Research Ethics Committee of King Faisal University (approval number: KFU-REC-2022-SEP-ETHICS1,329) to ensure the safety and protection of participants throughout the research process.

## Results

The results showed that there were 406 participants. Most of them (240, 59%) were female, while 166 (41%) were male. Approximately half of the participants (206, 51%) were aged less than 25 years, 115 (28%) were between 25 and 40 years old, 64 (16%) were between 41 and 55 years old, and 21 (5%) were over 55 years old. The majority (146, 61%) were single, 148 (36%) were married, seven (2%) were divorced, and five (1%) were widowed. Regarding education level, the majority (253, 62%) had a bachelor's degree, 103 (25%) had completed secondary school, 26 (6%) held an undergraduate diploma, 13 (3%) had completed lower school, and 10 (2%) had a postgraduate degree. Concerning occupational status, the majority (183, 45%) were students, 108 (26%) were employed, 48 (12%) were housewives, 33 (8%) were retired, and 37 (9%) were unemployed. As for monthly income, the majority (214, 53%) had an income of less than 3000 SR, 120 (25%) had incomes between 3000 and 10000 SR, 67 (17%) had incomes between 10000 and 20000 SR, and 23 (6%) had incomes exceeding 20000 SR. Regarding chronic diseases, the majority (329, 81%) reported having no chronic diseases, while 10 (2.5%) had hypertension, 18 (4.4%) had diabetes, 15 (3.7%) had dyslipidemia, three (0.7%) had heart diseases, and 31 (7.6%) had other diseases (Table 1).

**Table 1:** Demographic characteristics

Variables	N=406	%
Gender		
Male	166	41
Female	240	59
Age groups		
Less than 25 years	206	51
From 25-40 years	115	28
From 41-55 years	64	16
More than 55 years	21	5
Marital status		
Single	246	61
Married	148	36
Divorced	7	2
Widowed	5	1
Education level		
Lower school	14	3
Secondary school	103	25
Undergraduate diploma	26	6
Bachelor	253	62
Postgraduate	10	2
Occupation status		
Student	183	45
Housewife	48	12
Employed	105	26
Retired	33	8
Unemployed	37	9
Income		
Less than 3000 SR	214	53
From 3000-10000 SR	102	25
From 10000-20000 SR	67	17
More than 20000 SR	23	6
Chronic disease		
No diseases	329	81
Hypertension	10	2.5
Diabetes	18	4.4
Dyslipidemia	15	3.7
Heart diseases	3	0.7
Other	31	7.6

The results revealed that the knowledge level about AMI was 73.05%. The most knowledgeable item was "Sudden heart attack requires prompt treatment" (372, 91.6%), followed by "Have you ever heard about acute myocardial infarction?" (336, 90.1%), and then by "If you want to call an ambulance, do you know the phone number?" (350, 86.2%). This was followed by "Have you heard about the risk factors of acute myocardial infarction?" (262, 64.5%), "Do you know anyone who had acute myocardial infarction before?" (217, 53.4%), and "Have you ever received any information related to acute myocardial infarction?" (213, 52.5%) (Table 2).

**Table 2:** Knowledge about AMI

Items	Yes	No	I don't know	Level
Have you ever heard about acute myocardial infarction?	366	32	8	90.1%
	90.1%	7.9%	2%	
Do you know anyone who had acute myocardial infarction before?	217	156	33	53.4%
	53.4%	38.45%	8.1%	
Have you ever received any information related to acute myocardial infarction?	213	193	0	52.5%
	52.5%	47.5%	0%	
Sudden heart attacks require prompt treatment.	372	4	30	91.6%
	91.6%	1%	7.4%	
If you want to call an ambulance, do you know the phone number?	350	56	0	86.2%
	86.2%	13.8%	0%	
Have you heard about the risk factors of acute myocardial infarction?	262	144	0	64.5%
	64.5%	35.5%	0%	
Total				73.05

The results indicated that the majority of participants (301, 74.1%) had a good level of knowledge, while 105 (25.9%) had a poor level (Table 3).

**Table 3:** Distribution of knowledge level between participants

No	Score	Level	N	%
1	0-3	Poor	105	25.9
2	4-6	Good	301	74.1

The results revealed that the majority of participants (216, 53.2%) believed that if someone shows signs and symptoms of AMI, they should call an ambulance first, while 162 (39.9%) suggested taking the individual to the hospital. Only nine (2.2%) recommended calling their doctors, and two (0.5%) advised contacting their family. Additionally, two (0.5%) suggested trying to provide first aid, whereas 15 (3.7%) indicated they didn’t know (Table 4).

**Table 4:** The initial action for a person experiencing AMI

The action	N	%
Take them to the hospital	162	39.9
Call their doctors	9	2.2
Call an ambulance	216	53.2
Contact their family	2	0.5
Try to provide first aid	2	0.5
I don't know	15	3.7

The results indicated that the primary source of information related to AMI was the Internet (140, 28.9%), followed by healthcare professionals (113, 23.3%), books (79, 16.3%), media (69, 14.2%), TV (45, 9.3%), seminars (23, 4.7%), promotional leaflets (14, 2.9%), and lastly, family and friends (2, 0.4%) (Table 5).

**Table 5:** Primary sources of information related to AMI

Sources	N	%
Internet	140	28.90%
Healthcare professionals	113	23.30%
Books	79	16.30%
Media	69	14.20%
TV	45	9.30%
Seminars	23	4.70%
Promotional leaflets	14	2.90%
Family and friends	2	0.40%

The results indicated that the most recognized symptom of AMI was "sudden pain or discomfort in arms or hands" (287, 27.8%), followed by "sudden shortness of breath" (257, 24.9%), "sudden pain or discomfort in arms or shoulders" (175, 16.9%), "sudden pain or discomfort in the jaw, neck, or back" (118, 11.4%), "weakness or dizziness" (106, 10.3%), "sudden disturbance of vision in one or both eyes" (87, 8.4%), and "chest pain," "body relaxation and heaviness," and "pain between the abdomen and the chest cage," all at 1 (0.1%) (Table 6).

**Table 6:** Symptoms of AMI

Symptoms	N	%
Sudden pain or discomfort in arms or hands	287	27.8
Sudden disturbance vision in one or both eyes	87	8.4
Sudden pain or discomfort in arms or shoulders	175	16.9
Sudden shortness of breath	257	24.9
Sudden pain or discomfort in the jaw, neck, or back	118	11.4
weakness or dizziness	106	10.3
Chest pain	1	0.1
Body relaxation and heaviness	1	0.1
Pain between the abdomen and the chest cage	1	0.1
Total	1033	100

The results indicated that the most recognized risk factors for AMI were "obesity" (304, 12.3%) and "heart disease' (305, 12.3%), followed by "smoking" (301, 12.2%), "high blood pressure" (265, 10.7%), "high cholesterol" (215, 8.7%), "stress" (214, 8.6%), "lack of exercise" (175, 7.1%), "unhealthy diet" (174, 7%), "diabetes" (163, 6.6%), "alcohol" (138, 5.6%), "genetic" (102, 4.1%) and "atrial fibrillation" (101, 4.1%), and finally, "exercise" (9, 0.4%). Additionally, there were nine (0.4%) participants who indicated they didn't know (Table 7).

**Table 7:** Risk factors of AMI

Risk factors	N	%
Smoking	301	12.2
Obesity	304	12.3
Diabetes	163	6.6
Genetic	102	4.1
Unhealthy diet	174	7.0
Stress	214	8.6
High blood pressure	265	10.7
Alcohol	138	5.6
Atrial fibrillation	101	4.1
High cholesterol	215	8.7
Lack of exercise	175	7.1
Heart disease	305	12.3
Exercise	9	0.4
I don't know	9	0.4
Total	2475	100

The results indicated that the percentage of good knowledge was approximately equal among males, with 123 (74.7%), and females, with 177 (73.8%), showing no significant association ( = 0.046, P-value = 0.830). The highest percentage of good knowledge was observed among participants aged 41 to 55 years, with 51 (79.7%), demonstrating a significant association ( = 10.55, P-value = 0.014). Similarly, participants who were widowed exhibited the highest percentage of good knowledge, with 4 (80%), but there was no significant association ( = 1.841, P-value = 0.585). Among participants with a bachelor's degree, 195 (77.1%) demonstrated good knowledge, showing a significant association ( = 10.546, P-value = 0.032). Students also displayed a high percentage of good knowledge, with 143 (78.1%), and a significant association ( = 13.177, P-value = 0.010). Participants with a monthly income ranging from 3000 to 10000 SR had the highest percentage of good knowledge, with 84 (82.4%), yet there was no significant association ( = 6.792, P-value = 0.079). Lastly, participants with no chronic diseases exhibited a good knowledge percentage of 248 (75.4%), without any significant association ( = 1.396, P-value = 0.237) (Table 8).

**Table 8:** Distribution of knowledge level according to demographic characteristics *significant at 0.05

Variables	Categories	Knowledge level				Chi-square	p-value
		Poor		Good			
		N	%	N	%		
Gender	Male	42	25.3	124	74.7	0.046	0.830
	Female	63	26.3	177	73.8		
Age groups	Less than 25 years	47	22.8	159	77.2	10.55	0.014*
	From 25-40 years	34	29.6	81	70.4		
	From 41-55 years	13	20.3	51	79.7		
	More than 55 years	11	52.4	10	47.6		
Marital status	Single	58	23.6	188	76.4	1.941	0.585*
	Married	44	29.7	104	70.3		
	Divorced	2	28.6	5	71.4		
	Widowed	1	20	4	80		
Education	Lower school	8	57.1	6	42.9	10.546	0.032*
	Secondary school	26	25.2	77	74.8		
	Undergraduate diploma	10	38.5	16	61.5		
	Bachelor	58	22.9	195	77.1		
	Postgraduate	3	30	7	70		
Occupation	Student	40	21.9	143	78.1	13.177	0.010*
	Housewife	11	22.9	37	77.1		
	Employed	24	22.9	81	77.1		
	Retired	13	39.4	20	60.6		
	Unemployed	17	45.9	20	54.1		
Income	Less than 3000 SR	64	29.9	150	70.1	6.792	0.079
	From 3000-10000 SR	18	17.6	84	82.4		
	From 10000-20000 SR	15	22.4	52	77.6		
	More than 20000 SR	8	34.8	15	65.2		
Chronic	No	81	24.6	248	75.4	1.396	0.237
	Yes	24	31.2	53	68.8		

## Discussion

This study aimed to comprehensively assess public awareness of AMI in Alahssa, Saudi Arabia. The findings shed light on the knowledge levels regarding AMI, awareness of risk factors and symptoms, and the actions individuals believe should be taken during an AMI event. The study revealed a moderate overall knowledge level about AMI, with over two-thirds of participants demonstrating some level of awareness. Notably, three-quarters of the participants exhibited a good knowledge level, while one-quarter had a poorer knowledge level. When it comes to responding to an AMI event, more than half of the participants correctly identified that the first step should involve calling an ambulance. Additionally, participants primarily relied on the Internet and healthcare professionals as their main sources of AMI-related information. In terms of recognizing symptoms of AMI, "sudden pain or discomfort in arms or hands" emerged as the symptom most commonly known among participants. Moreover, in their understanding of AMI risk factors, participants largely recognized "obesity" and "heart disease" as significant factors. Nonetheless, the study identified noteworthy variations in knowledge levels based on demographic characteristics.

The current study found that 73.05% of participants had some level of knowledge about AMI. This level of awareness is consistent with global trends, where two studies in America and Saudi Arabia have reported awareness levels ranging from 60% to 80% [12-13]. It indicates that awareness of AMI is a concern across various regions and populations. Our study observed that 74.1% of participants had a good knowledge level, while 25.9% had a poor knowledge level. This distribution is similar to findings in other studies in America and Saudi Arabia that also identified a knowledge gap in a significant portion of the population [12-13]. This suggests that efforts to improve awareness should be tailored to address the needs of those with poor knowledge.

The current study revealed that 53.2% of participants believed that calling an ambulance should be the first action when someone shows signs and symptoms of AMI. This aligns with international guidelines that emphasize the importance of immediate medical attention [8]. However, there is still room for improvement, as a significant percentage considered alternative actions. Similar findings have been reported in a study in South Korea [9]. This underscores the need for public education on the correct response to AMI symptoms. Moreover, in our study, participants identified the Internet (28.9%) and healthcare professionals (23.3%) as the primary sources of information about AMI. These findings are consistent with studies in South Korea and Germany, which also highlighted the Internet and healthcare providers as key sources of AMI information [14,15]. It reflects the role of digital media and healthcare providers in disseminating information about AMI.

The current study revealed that the most recognized symptom of AMI among participants was "sudden pain or discomfort in arms or hands" (27.8%). These findings are in line with the international recognition of chest pain and discomfort as a hallmark symptom of AMI [8]. However, the knowledge levels of other symptoms were comparatively lower, indicating a need for increased awareness of a broader range of symptoms. Additionally, we found that "obesity" and "heart disease" were the most recognized risk factors for AMI (12.3%). These results are consistent with two studies conducted in America and Saudi Arabia that have also identified these risk factors as commonly recognized [12-13]. Nonetheless, there is room for improvement in awareness of other significant risk factors like smoking and high blood pressure.

Our study identified significant variations in knowledge levels based on demographic characteristics. For instance, participants aged 41 to 55 years exhibited the highest knowledge level (79.7%), and those with a bachelor's degree showed a high percentage of good knowledge (77.1%). These findings align with research in different regions, which often identifies age and educational level as factors influencing AMI awareness [14,16]. Tailored interventions should consider these demographic variations to effectively target specific groups.

Limitations

This study relied on an online survey distributed through social media platforms, which may introduce a sampling bias. Individuals without Internet access or who do not use social media may be underrepresented in the sample. The study collected data based on participants' self-reports, which are subject to recall bias and may not always reflect their actual knowledge or behavior accurately. The study was conducted in a specific region of Saudi Arabia (Alahssa), limiting its generalizability to other areas of the country or different cultural contexts. While efforts were made to ensure clarity in the questionnaire, language barriers may have affected participants' understanding and responses, potentially impacting the data's accuracy. The cross-sectional design captures data at a single point in time, preventing the assessment of changes in awareness and knowledge over time. Longitudinal studies could provide more insights into this aspect.

Recommendations

Future research should employ a more diverse data collection strategy, including in-person interviews and telephone surveys, to reach a broader spectrum of the population and reduce sampling bias. Conducting longitudinal studies to track changes in AMI awareness and knowledge over time would provide a better understanding of the effectiveness of awareness campaigns and educational initiatives. To address language barriers, researchers should consider providing questionnaires in multiple languages commonly spoken in the region to ensure inclusivity and accurate responses. By addressing these limitations and implementing these recommendations, future studies and initiatives can contribute to improved AMI awareness and outcomes in Saudi Arabia and potentially serve as a model for other regions.

## Conclusions

This cross-sectional study has provided valuable insights into AMI awareness among the general population in Alahssa, Saudi Arabia. The findings emphasize the significance of enhancing public knowledge regarding AMI symptoms and the appropriate actions to take during an AMI event. The study revealed a moderate overall knowledge level about AMI, with the majority of participants demonstrating awareness of key AMI-related aspects. Notably, three-quarters of the participants exhibited a good knowledge level, indicating a substantial understanding of AMI. In conclusion, these findings underscore the necessity of targeting specific demographic groups, considering factors such as age, education, and occupation, through educational campaigns and interventions. It is crucial to improve awareness and knowledge of AMI in the general population of Alahssa, Saudi Arabia. Additionally, ensuring that accurate information about AMI and its risk factors is readily accessible through healthcare institutions and digital platforms will contribute to better knowledge dissemination. Furthermore, healthcare professionals should receive training in effectively communicating AMI-related information to patients and the public, serving as reliable sources of information. These collective efforts will ultimately lead to more timely and effective responses to this life-threatening condition, significantly improving patient outcomes and reducing the impact of AMI on the population.
